# Sex Differences in Maintaining the Requested Handgrip Force Enhanced by Auditory or Visual Feedback

**DOI:** 10.3390/ijerph17145158

**Published:** 2020-07-17

**Authors:** Jacek Tarnas, Rafał Stemplewski, Piotr Krutki

**Affiliations:** 1 Department of Physical Education and Lifelong Sports, Poznan University of Physical Education, Królowej Jadwigi 27/39, 61-871 Poznań, Poland; 2 Department of Physical Activity Sciences and Health Promotion, Poznan University of Physical Education, Królowej Jadwigi 27/39, 61-871 Poznań, Poland; stemplewski@awf.poznan.pl; 3 Department of Neurobiology, Poznan University of Physical Education, Królowej Jadwigi 27/39, 61-871 Poznań, Poland; krutki@awf.poznan.pl

**Keywords:** auditory stimulus, visual stimulus, motor learning, force control

## Abstract

Thus far, the differences in effect of auditory or visual feedback in motor learning have presented results derived from mixed groups and sex differences have not been considered. However, perception and processing of auditory stimuli and performance of visual motor tasks appear to be sex-related. The purpose of this study was to investigate the learning of the simple motor task of maintaining a requested handgrip force in separate male and female groups. A total of 31 volunteers (15 males, 16 females) were randomly assigned to one of four experimental groups with defined sex and training conditions (audio or visual feedback). Participants performed training sessions over a period of six days, for which auditory or visual feedback was provided, and the effectiveness of both types of signals was compared. The evident learning effect was found in all groups, and the main effect of sex was significant among visual groups in favor of the males (*p* < 0.05). On the other hand, the main effect of feedback conditions was found to be significant among females, beneficially in the case of auditory displays (*p* < 0.05). The results lead to the conclusion that an equal number of males and females in mixed experimental groups may be supportive to obtain reliable results. Moreover, in motor-learning studies conducted on females only, a design including auditory feedback would be more suitable.

## 1. Introduction

Motor control and motor learning can influence the quality of human movement and are crucial components during sport skill acquisition, physical education and rehabilitation, work, and other forms of exercise. It is possible to accelerate these processes using intrinsic feedback and by providing extrinsic (augmented) feedback [1]. Numerous studies have been conducted to develop strategies to enhance motor performance, in which augmented feedback was essential [2]. Schmidt and Wrisberg [3] defined augmented feedback as information that cannot be elaborated without an external source; thus, it should be provided by a coach or a display. Such information can be provided in different single or combined modalities via various displays, e.g., visual, auditory, and/or haptic [4]. In recent research, the role of auditory information on perceptual-motor processes has gained increased interest [5]. Close interaction between the auditory and motor areas of the brain, and the importance of auditory information for movement execution, control, and learning was demonstrated. Interestingly, the auditory and motor systems are co-activated even when only movement or sound is produced [6,7]. However, there are reports indicating that sex differences exist in the perception and processing of auditory stimuli. Goldstein et al. [8] suggested that auditory working memory tasks induce different activation patterns in the auditory regions in males and females. McFadden [9] reported some differences in auditory perception, e.g., females had stronger optoacoustic emissions than males. This phenomenon has been found in newborns, which suggests that it is not connected to environmental experience [10]. Additionally, females are more sensitive to a given physical range of tones than males [11]. Ruytjens et al. [12] found sex differences in brain activity at the level of primary sensory cortex when comparing music and noise with the use of PET (positron emission tomography). Furthermore, it was also suggested that placing males and females in one group in auditory neuroimaging studies may obscure or bias the results [8].

Sex differences in task effectiveness when auditory feedback has been provided during motor learning have not been adequately investigated. Previous studies using augmented auditory feedback during sport skill acquisition [13] and physical rehabilitation [14] have not made adequate intersexual comparisons.

Therefore, the main aim of this study was to analyze sex differences in the effectiveness of learning the simple motor task of maintaining a requested handgrip force during daily training sessions over a period of six days. The trials were performed on separate male and female groups, for which auditory or visual feedback was provided, and the effectiveness of both types of signals were compared.

## 2. Materials and Methods

### 2.1. Participants

We recruited a total of 31 volunteers (15 males, 16 females). All participants were right-handed, with no physical impairments or deficits in hearing and vision. Volunteers were randomly assigned to one of four experimental groups with defined training conditions separately for both sexes (audio or visual feedback): audio males (AM; *n* = 7, 21–23 years old, mean age = 22.7 ± 0.8 years), visual males (VM; *n* = 8, 21–28 years old, mean age = 24.1 ± 1.9 years), audio females (AF; *n* = 8, 21–24 years old, mean age = 22.5 ± 0.8 years), and visual females (VF; *n* = 8, 21–22 years old, mean age = 22.0 ± 1.9 years). All the participants gave their written consent to participate in this study. All procedures followed the principles outlined in the Declaration of Helsinki, and the study was approved by the Local Bioethical Committee (no. 198/16).

### 2.2. Instrumentation

Data on requested handgrip force were collected with the Baseline^®^ digital hydraulic hand dynamometer with transducer (Fabrication Enterprises, Inc., New York, NY, USA), with a sampling rate of 50 Hz. The electrical signal generated by the transducer was transmitted to a computer and processed by a custom-made program.

The continuous audio signal (sine wave 440 Hz) was generated by the computer routed to headphones (Technics RP-F300, Panasonic Corporation, Japan). The audio volume was set to a comfortable decibel level for an individual (≈40 dB). The continuous visual signal was provided as a white circle (⌀ = 13 cm) on a dark background on a 20” computer monitor, with a viewing area of 1600 × 900 pixels and a dot pitch width of ≈0.28 mm (Flatron W2043T-PF; LG:Seoul, Korea).

### 2.3. Protocol

The maximum voluntary contraction (MVC) and performance tests (50% of MVC) were the principal tasks performed during the experiment. We instructed participants to sit on a chair 50 cm away from a computer monitor. The tested limb (dominant) was slightly flexed at the elbow joint, resulting in an approximate 135° angle between the arm and forearm. The palm and fingers were clasped around the handle. The MVC was calculated as an average of three trials (peak forces) of the maximum forearm strength [15]. The performance test was calculated as the average of the force level during a 6-s trial.

**Figure 1:**
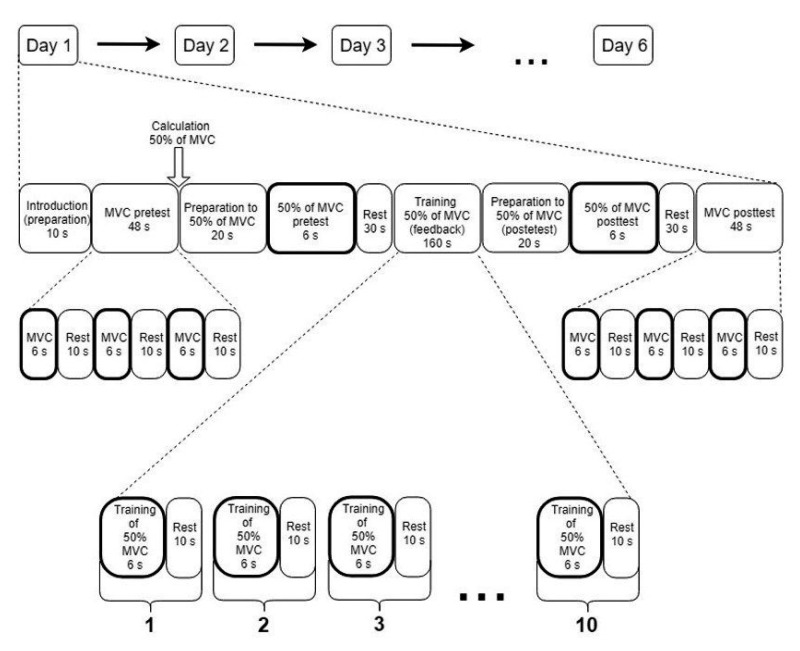
The experimental protocol of the training program performed each day by all the groups. MVC: maximum voluntary contraction.

The experiment was performed one time per day during six consecutive days at approximately the same time and with an equal training program each day (Figure 1). We asked participants to follow the prompts displayed on the computer screen, on which necessary commands and information about the time remaining until subsequent tasks were given. At the beginning of the experiment, all individuals performed three MVCs (pre-test). After a rest period, participants were instructed to execute the performance pre-test. The result of the pre-test was the performance error (PE) for an individual, which was calculated as the size of the deviation from the expected value (50% of MVC: pre-test) as expressed in percentages.

Following 30 s of rest, the participants performed ten consecutive training trials for maintaining the target, i.e., 50% of MVC (6 s), with 10 s rest between each trial. The individuals received feedback on their performance via auditory or visual feedback, at a range of ±20 N from the target. The ranges were stable for males and females (between 272.2–312.2 N and 130.2–170.2 N, respectively), regardless of audio and visual groups. Under the auditory feedback conditions, once the individual matched the requested force range, a pure tone was perceived in both ears. Likewise, under the visual feedback conditions, the individuals matched the target force by triggering a display of a white circle on a dark background. The aim for participants was to maintain requested force in previously calculated and set range. In the case of being out of range, there was no auditory or visual feedback, and it was interpreted as an error. After the training session and successive rest, the performance post-test was performed. The result of the post-test for each individual was the PE (analogically to pre-test). At the end of the experiment, following 30 s of rest, individuals performed three MVCs (post-test) for the determination of their maximum forearm strength as a reference for PE post-test. The participants were not informed about the results of the performance pre-tests and post-tests during the entire 6-day period of the experiment.

### 2.4. Statistical Analysis

Statistical analyses were done via Dell Statistica (data analysis software system), version 13 (TIBACO Software Inc.: Palo Alto, CA, USA). Differences were considered statistically significant at *p* < 0.05. Two-way analysis of variance (ANOVA) was employed to compare the differences between the values of the PE between tested groups. The analysis with two levels for the first factor (within-subject factor “session”: the pre-test and post-test) and six levels for the second factor (within-subject factor “time”: the first to the sixth day of training) separately for males and females (for both audio and visual conditions) was used. For comparison between males and females, a two-way ANOVA with two levels for the first factor (between-subject factor “sex”: male, female) and six levels for the second factor (within-subject factor “time”: the first to the sixth day of training) was conducted separately for the pre-test and post-test (for both audio and visual conditions). For a comparison between the audio and visual feedback conditions, a two-way ANOVA with two levels for the first factor (between-subject factor “feedback”: audio, visual) and six levels for the second factor (within-subject factor “time”: the first to the sixth day of training) was run separately for the pre-test and post-test (for both males and females). The differences between the groups in PE values at the baseline in the pre-test and post-test (between sexes, separately for audio and visual conditions, as well as between conditions, separately for males and females) were calculated via the Bonferroni detailed post-hoc tests.

For interaction effects, the eta-squared (*η*^2^) effect size was calculated. The effect size indicates the percent of the variance explained by the particular effects of the dependent variable. It was also used to calculate the power of significant effects. Differences between males and females were analyzed on a basis of the main effects of the “sex” factor.

## 3. Results

**Figure 2:**
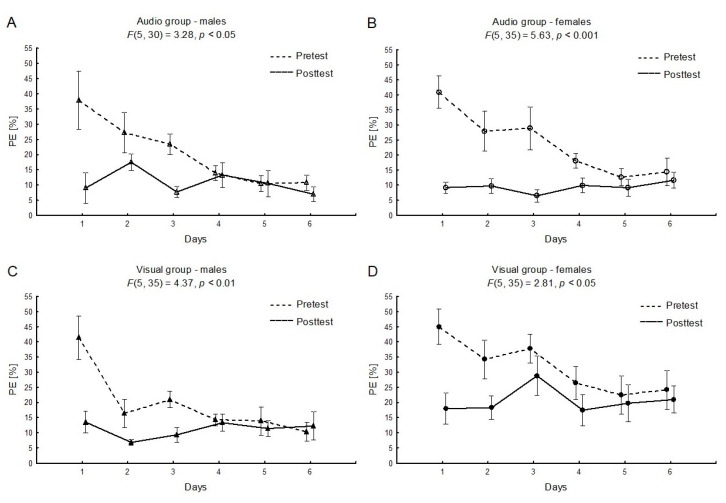
The mean relative values and standard error of measurements of the performance error (PE) for the “session × time” factor (the pretest vs. the posttest), separately for males and females, for the audio (**A**,**B**) or the visual feedback (**C**,**D**).

There were no sex differences between the groups for PE values at the baseline in pre-tests and post-tests (between sexes, separately for audio and visual conditions, as well as between conditions, separately for males and females). The two-way interaction effect “session × time” was significant for females and males with respect to both auditory and visual feedback conditions (AM: F_(5, 30)_ = 3.28, *η*^2^ = 0.35, *p* < 0.05, power statistic = 0.83; AF: F_(5, 35)_ = 5.63, *η*^2^ = 0.45, *p* < 0.001, power statistic = 0.98; VM: F_(5, 35)_ = 4.37, *η*^2^ = 0.38, *p* < 0.01, power statistic = 0.94; VF: F_(5, 35)_ = 2.81, *η*^2^ = 0.29, *p* < 0.05, power statistic = 0.77: see Figure 2 (A, B, C, and D, respectively). Our results indicated a significant decrease in the PE difference between the pre-test and post-test in following days.

**Figure 3:**
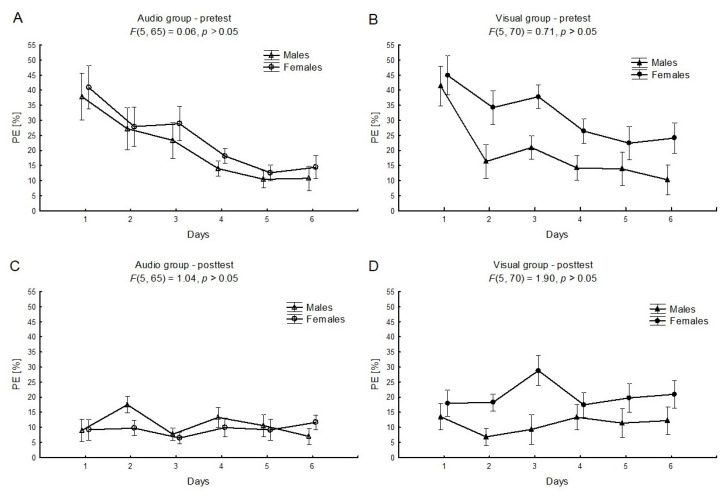
The mean relative values and standard errors of the performance error (PE) for the “sex × time” factor (males vs. females), separately for the pretest and the posttest, for the audio (**A**,**B**) or the visual feedback (**C**,**D**).

The two-way interaction effects “sex × time” for audio and visual feedback conditions in pre-tests (Figure 3A,B) were not significant (F_(5, 65)_ = 0.06, *η*^2^ = 0.01, *p* > 0.05; F_(5, 70)_ = 0.71, *η*^2^ = 0.05, *p* > 0.05, respectively), which indicated a similar trend for a decrease in the PE among males and females. On the other hand, the main effect for the “sex” factor for the visual feedback conditions in pre-tests was statistically significant (F_(1, 14)_ = 7.38, *η*^2^ = 0.35, *p* < 0.05, power statistic = 0.71). Females had higher levels of the PE than males (Figure 3B). There were no significant interaction effects for “sex × time” nor main effects for the “sex” factor for the audio and the visual feedback conditions in post-tests (Figure 3C,D).

**Figure 4:**
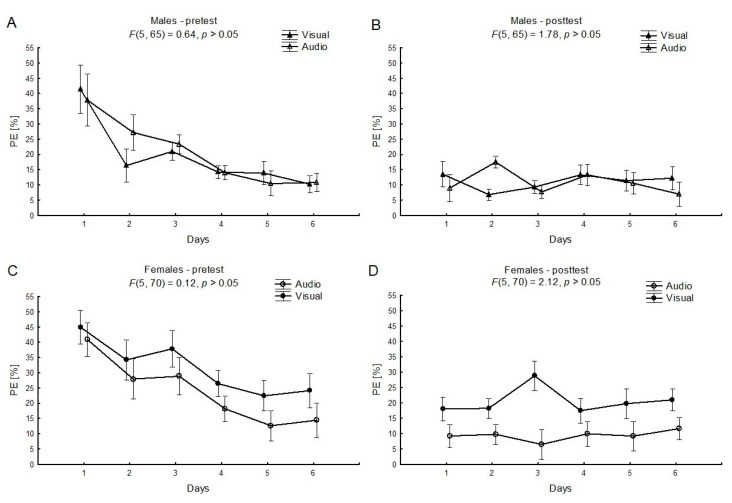
The mean relative values and standard errors of the performance error PE [%] for the “feedback × time” factor (the audio vs. the visual feedback), separately for the pretest and the posttest, for males (**A**,**B**) or females (**C**,**D**).

Moreover, there was no significant interaction effect in the learning process over the six days (Figure 4) for the audio and visual feedback conditions (“feedback × time”), separately among males and females. On the other hand, the main effect for the “feedback” factor within females in post-tests was statistically significant (F_(1, 14)_ = 5.84; *η*^2^ = 0.29, *p* < 0.05, power statistic = 0.61). Female group practicing under visual feedback conditions had higher levels of PE than females under audio feedback conditions (Figure 4D). 

## 4. Discussion

The major finding of this study was the clear learning effect (see Figure 2) during the six-day training period for a simple motor task, which was distinctly visible for both males and females in the decrease of pretest PE values. This effect reflected the second phase of learning, during which errors in detection/correction mechanisms were improved [3]. In our study, the tendency towards enhancement of the PE was noticeable in all groups from the first to the fourth day in the pre-tests, whereas in the last two days of the experiment, the PE in pre-tests remained stable. Participants tended to perform the post-tests (without feedback) at comparable levels of PE after each training session. The improvement in the hand force production task would probably be more efficient in combination with terminal feedback during the same trial or when no-feedback trials are added [16].

There was no significant interaction effect in the six-day learning process (see Figure 3) between males and females, independently of feedback conditions. On the other hand, the main effect of sex was significant among visual groups, in favor of the males. There are premises that males perform better than females in visual motor tasks related to the control of muscle strength in precise hand movements. Thorson at al. [17] concluded that the sex differences should not be ignored in training programs for medical students, when using a simulation laparoscopic trainer. Therefore, when designing research aimed at analyzing interactions between males and females in the field of feedback in motor learning, the sex independence of the motor tasks should be considered attentively.

Maintaining handgrip requires an isometric force in which the inertial properties of a limb movement and the contraction speed of a muscle are minimized, possibly providing optimal conditions with which to investigate the role of different types of feedback on the output of the neuromuscular system. On the other hand, it is well established that sex is the major factor differentiating hand grip [18]. Repetitions of the requested 50% of MVC in the current study have certain endurance demands. Nevertheless, similar static (50% of MVC) handgrip endurance times were reported between males and females sport climbers, although the male climbers exhibited a greater maximum handgrip strength [19]. Moreover, males and females exhibited also a similar exercise tolerance during repeated forearm muscle contractions [20], and no significant differences existed between the genders in measures of relative handgrip endurance [21]. In the light of mentioned outcomes, the task applied in the current research appears to be appropriate for tests analyzing sex differences.

In our study, the main effect of the sex factor between visual groups in favor of males in pre-tests was observed. Nevertheless, discussion of this phenomenon has some limitations. If the auditory and visual feedback designs were rarely conducted for sexually homogeneous groups, they were usually unique and specific. For example, the effectiveness of augmented auditory feedback on the performance and learning of precision shooting was tested among male soldiers, who were conscripts recruited from the Finnish army [22]. Highly experienced gymnastic male groups participated in a research study in which the effectiveness of concurrent auditory feedback on segmental body alignment during the circle movement performed on a pommel horse was tested [23]. However, since the efficiency of an auditory display is task-dependent [24], the revealed results need to be considered individually. Effectiveness of the auditory or visual feedback in a distinct condition was commonly assessed in mixed experimental groups, with balanced (but not always) participation of males and females. For example, during the acquisition of a new bimanual coordination pattern, an application of the augmented visual feedback showed faster progress in comparison to an auditory one, but performance deteriorated significantly after the external presentation was withdrawn [25]. It is most likely that the participants became dependent on the augmented visual feedback. In contrast, the auditory group preserved its equally good performance level even after the removal of external support and showed less dependency on the feedback. These results were explained by indicating a growing neural activity in sensory-specific areas of the brain. Decreased neural activity observed in the audio group was explained by the authors as a development of an independent control strategy. Similar results were reported by Chiou and Chang [26]. The authors suggested that learning of a bimanual coordination pattern with a visual feedback was vulnerable to feedback removal, while learning with an auditory feedback seemed to persevere to a greater extent. Nevertheless, it was highlighted that learning process, as well as the retention performance, depends more on the structure of information (continuous Lissajous feedback to the visual group and discrete rhythmic feedback to the auditory group) than on the feedback type provided during practice.

It was also demonstrated that there was no significant interaction effect over the six-day learning process (see Figure 4) between audio and visual feedback conditions within each sex. Nevertheless, the main effect of the feedback in post-tests was found to be significant among females, in favor of auditory displays. Generally, in the case of maintaining the requested handgrip force with a use of different feedback modalities, females revealed higher PEs (see Figure 4), and visual display was significantly less effective among females. As mentioned previously, the nature of the task requirements may play a role in determining an advantage. In our study, practicing with the auditory display allowed females to reach performance levels similar to achievements of males, and presumably, some abilities of females, such as strong auditory perception [9] and sound discrimination [27], were beneficial.

Generally, audition has beneficial effects on perception accuracy, reproduction and regulation of movement patterns [28]. The findings appear to be substantial in sport, due to the high temporal resolution of hearing and unrestrained movement [29]. In our study, we applied continuous audio and visual signals during execution of a simple motor task and therefore a transfer of the conclusions to complex task learning in sports and rehabilitation may be limited [30]. On the other hand, the auditory feedback guided the focus towards a specific aspect of the movement [31,32]. An acoustic signal can reflect different features of a given movement (e.g., timing, force, duration and pressure). Technological progress in development of electronic tools enables us to improve motor skills in a variety of sports [33], as well as to enhance motor learning in rehabilitation [34].

In summary, this study demonstrated a significant learning effect in maintaining the requested handgrip force, which was enhanced during consecutive days of training by either auditory or visual feedback. Hence, the task design appeared to be appropriate for tests in which sex differences are analyzed. The limitation of the study was that there was no retention test after longer period of time. In our study from the second day, each pretest (50% of MVC) would be treated as the retention test, after 24 h of rest. However, an additional test, e.g., after the one week, certainly would bring valuable information. Secondly, the tasks performed during the experiment were only simple ones and the groups were relatively small. On the other hand, very high effect sizes and power statistics were noted (*η*^2^ = 0.37, power statistics = 0.88 on average, respectively) for interaction effects (“session × time”). In fact, power statistics for the main effect of the “sex” factor were slightly lower (power statistics = 0.71), but its effect size was still high (*η*^2^ = 0.35). Despite a limited sample size, the study demonstrated a tendency of lower effectiveness of the visual feedback among females. Therefore, these findings may have important consequences for the planning of future research on similar topics and indicate the necessity of paying attention to the sex factor.

## 5. Conclusions

Our results lead to the conclusion that an equal number of males and females in mixed experimental groups seems to be supportive to obtain reliable results, especially if a visual factor is applied as a feedback stimulus. On the other hand, in studies on athletes’ performances are conducted on sexually homogenous groups, due to the specific characteristics of different sports disciplines, a design of auditory feedback seems to have a more specific effect for female groups. Nevertheless, for the optimization of augmented feedback in sports and rehabilitation, further studies on sex differences need to be conducted on complex motor tasks.
